# Global estimates of the number of pregnancies at risk of malaria from 2007 to 2020: a demographic study

**DOI:** 10.1016/S2214-109X(22)00431-4

**Published:** 2022-12-13

**Authors:** Valentina Reddy, Daniel J Weiss, Jennifer Rozier, Feiko O ter Kuile, Stephanie Dellicour

**Affiliations:** a Department of Clinical Sciences, Liverpool School of Tropical Medicine, Liverpool, UK; b Telethon Kids Institute, Perth Children's Hospital, Perth, WA, Australia; c Faculty of Health Sciences, Curtin University, Perth, WA, Australia

## Abstract

**Background:**

The most recent global estimates of the number of pregnancies at risk of *Plasmodium falciparum* and *Plasmodium vivax* malaria infection are from 2007. To inform global malaria prevention and control efforts, we aimed to estimate the global distribution of pregnancies at risk of malaria infection from 2007 to 2020.

**Methods:**

We used estimates from the Malaria Atlas Project on the total population living in areas of *P falciparum* and *P vivax* transmission, combined with country-specific demographic data on women of reproductive age, fertility rates, induced abortions, and stillbirths, to derive the annual number of pregnancies overall, by parasite species, and by endemicity strata from 2007 to 2020. The definition of endemicity strata was based on the parasite point prevalence in individuals aged 2–10 years for *P falciparum* and 1–99 years for *P vivax*. We also did a sensitivity analysis in which we considered most of sub-Saharan Africa endemic for *P vivax*.

**Findings:**

In 2020, 121·9 million pregnancies occurred in malaria transmission areas, resulting in an estimated 70·9 million (58·1%) livebirths. The total number of pregnancies at risk of malaria was 52·9 million in the WHO South-East Asia (SEARO) region, 5·1 million in the Western Pacific (WPRO) region, 46·1 million in the Africa (AFRO) region, 11·1 million in the Eastern Mediterranean (EMRO) region, and 6·7 million in the Americas (AMRO) region. Between 2007 and 2020, pregnancies in areas of *P falciparum* transmission declined by 11·4% globally, despite an overall 7·0% increase in pregnancies, representing a decrease of 100·0% in the WHO Europe (EURO) region, 52·6% in WPRO, 51·5% in AMRO, 23·9% in EMRO, and 17·2% in SEARO, and a 25·4% increase in AFRO. Pregnancies in *P vivax* transmission areas fell by 42·8%, representing a decrease of 100·0% in EURO, 89·8% in WPRO, 48·4% in AMRO, 32·4% in EMRO, and 10·0% in SEARO, and a 25·8% increase in AFRO. Our sensitivity analysis suggests that the number of pregnancies at risk of *P vivax* infection could be seven-fold higher for AFRO if the whole of sub-Saharan Africa was considered endemic for *P vivax*.

**Interpretation:**

Between 2007 and 2020, substantial declines in the number of pregnancies at risk of malaria were seen globally. However, in AFRO, 25·4% more pregnancies were at risk of *P falciparum* or *P vivax* malaria than in 2007. This increase in the number at risk in AFRO comes despite the decline in malaria rates due to the rapidly rising population and the corresponding number of pregnancies in endemic areas. These estimates should guide priority setting for resource allocation to control malaria in pregnancy.

**Funding:**

Bill & Melinda Gates Foundation and Telethon Trust.

## Introduction

Malaria infection carries serious risks for pregnant women, their fetuses, and their newborn babies. Pregnant women are at higher risks of malaria-related complications and death than aged-matched women who are not pregnant.^1^, ^2^, ^3^ Malaria in pregnancy can lead to maternal anaemia, fetal loss, preterm birth, low birthweight, and cognitive-developmental delays in infants.^4^, ^5^, ^6^ Of the five malaria parasite species that infect humans, *Plasmodium falciparum* and *Plasmodium vivax* are the major contributors to adverse maternal and pregnancy outcomes.^7^ In 2020, malaria in pregnancy was estimated to result in 819 000 low-birthweight babies in sub-Saharan Africa.^8^ WHO recommends prompt diagnosis and treatment of malaria in pregnancy as well as malaria prevention with long-lasting insecticide-treated nets and intermittent preventive treatment with sulfadoxine–pyrimethamine from the second trimester onwards in malaria-endemic areas.^9^

The most recent global estimates of the number of pregnancies at risk of malaria for *P falciparum* and *P vivax* are from 2007.^10^ These estimates are likely to have changed between 2007 and 2020, reflecting population growth and intensified malaria control efforts.^11^, ^12^, ^13^ In this study, we aimed to provide current estimates of the global distribution of pregnancies at risk of malaria for 2020 and provide data on trends since 2007 to help to inform key stakeholders to support targeted and equitable access to malaria prevention and treatment for pregnant women globally.

**Research in context**

**Evidence before this study**

Mapping pregnancies at risk of malaria is crucial for informing control strategies and targeting interventions for malaria in pregnancy. The most recent update on the number and global distribution of pregnancies at risk of malaria is for 2007, which showed that the number of pregnancies at risk in the Asia-Pacific region was much higher than in other regions. However, new estimates are needed to reflect population growth, changes in fertility rate, and major reductions in malaria transmission since 2007. We searched PubMed and Google Scholar for articles related to quantifying the number of pregnancies at risk of malaria published between Jan 1, 2010, and Sept 29, 2022, using the search terms “((malaria OR Plasmodium OR falciparum OR vivax) AND (Pregnan* OR antenatal) AND (risk) AND humans[MeSH])”, with no language restrictions. No relevant publications were identified. The WHO World Malaria Report shows the number of pregnancies resulting in livebirths at risk of malaria in Africa only. This manuscript expands these estimates beyond Africa to all pregnancies at risk of malaria globally, including those resulting in spontaneous pregnancy loss and induced abortions.

**Added value of this study**

This study provides the most up-to-date estimates of the global distribution of pregnancies at risk of malaria. The study is based on improved estimates of populations at risk of malaria with substantially expanded data sources for *Plasmodium falciparum* and *Plasmodium vivax* prevalence relative to previous 2007 estimates by the Malaria Atlas Project. It is also the first report of time trends on the changing pattern of pregnancies at risk of malaria between 2007 and 2020 by geographical regions, endemicity levels, and parasite species. The study estimates a 28·8% decrease in pregnancies at risk of *P falciparum* or *P vivax* malaria despite a 7·0% increase in the number of pregnancies globally, reflecting the shrinking map of malaria transmission.

**Implications of all the available evidence**

The findings highlight the gains made in the Asia-Pacific regions since 2007. The increasing number of pregnancies at risk of either *P falciparum* or *P vivax* malaria infection in sub-Saharan Africa reflects rapid population growth, even as these areas are progressing towards controlling and eliminating malaria. These data provide valuable information for researchers, policy makers, and programme managers to allocate targeted resources to control malaria in pregnancy, monitor the effect of malaria control efforts, and inform priority areas for research.

## Methods

### Data sources

We used model-derived estimates from the Malaria Atlas Project (MAP), a WHO collaborating centre for geospatial malaria monitoring. Annual country-level estimates of the population at risk of *P falciparum* or *P vivax* transmission were derived from MAP for 2007 to 2020 by WHO malaria endemicity strata. We based endemicity class on the parasite prevalence rate in the population of individuals aged 2–10 years for *P falciparum* and 1–99 years for *P vivax*.

MAP provides estimates of the total population living within the global limits of *P falciparum and P vivax* transmission using surveillance data on the number of malaria cases, nationally representative cross-sectional surveys, and satellite imagery data of environmental predictors of the prevalence of *Anopheles* mosquitoes.^14^ MAP uses two modelling approaches based on the reliability and availability of data within endemic countries. For 36 high-burden countries in Africa, high-resolution (5 × 5 km) estimates of parasite rate for *P falciparum* were generated by MAP using a geostatistical modelling framework. The parasite rate model used household surveys as the response variable and environmental and malaria intervention datasets as predictor variables.^11^, ^12^ For the remaining malaria-endemic countries, MAP uses routinely reported surveillance data as the response variable after adjusting for the following factors for countries that are not in the elimination phase: treatment-seeking rates, cases treated outside of government facilities, under-reporting, and presumptive diagnosis. Missing years were imputed within a spatial disaggregation model to create high-resolution maps of parasite rate.^11^ This second method was used to create maps for both *P falciparum* and *P vivax*. For the high-burden African countries, the maps of *P v*ivax prevalence were created using the high-resolution *P falciparum* parasite rate maps and the proportions of *P vivax* cases using reported country-specific and year-specific ratios of *P vivax* to *P falciparum.*^12^

MAP's current approach to estimating the total population at risk for malaria is based on a combination of environmental datasets, all available routine survey data, and WHO endemicity status. This approach results in transmission limits that change over time as regions eliminate malaria or oscillate between reporting no and some malaria cases across annual surveys. To avoid contradicting routine surveillance data, MAP's parasite rate maps consider populations at risk in administrative units that reported malaria cases that year. Two exceptions to the previous statement are pixels that are set to zero malaria cases due to environmental suitability for *Plasmodium* and pixels set to no cases based on lower-level (ie, smaller) administrative subunits that reported no cases by routine surveillance in the same year. A third exception relates to Africa, as MAP only considers *P vivax* to be endemic in the horn of Africa despite evidence indicating that there might be *P vivax* transmission elsewhere in sub-Saharan Africa.^15^ To address this contradiction, we included a sensitivity analysis in which we considered most of sub-Saharan Africa endemic for *P vivax*.

The total population at risk estimates were split into endemicity strata data on the basis of the prevalence maps. Hypoendemic transmission was defined as areas with a parasite prevalence of 10% or less, mesoendemic as higher than 10–50%, hyperendemic as higher than 50–75%, and holoendemic as higher than 75%.^16^ Because the parasite rate model predicts a range of malaria values for each pixel, some pixels fall within multiple endemicity classes. As a result, the population at risk counts might not perfectly align with the sum of the people living within all endemicity classes as presented for the overall population at risk by species.

Uncertainty intervals (UIs) were calculated by MAP for estimates of population at risk stratified by species and endemicity level only. The 95% UIs for the malaria endemicity classes were created by processing a set of 100 realisations (ie, parasite rate maps) drawn from the posterior distribution of the geostatistical model) for each year. This process generated 100 estimates for the population at risk within each class for each country each year. From these 100 estimates, we derived the mean and upper UIs as 98th ranked estimates and lower UIs as 3rd ranked estimates. UIs could not be derived for overall risk estimates (*P falciparum, P vivax*, and risk of either or both species) because the spatial extent of malaria endemicity was fixed according to routine surveillance data.

### Data analysis

We calculated the annual estimates of pregnancies in each malaria-endemic country as the sum of the number of livebirths, induced abortions, and spontaneous pregnancy loss (including miscarriages and stillbirths) using different data sources for livebirths, stillbirths, and induced abortions.

For annual number of livebirths for each malaria-endemic country, we used country-specific total fertility rate (TFR) data (which is defined as the sum of age-specific fertility rates) and demographic data of the number of women of reproductive age (15–49 years as per the WHO definition^17^) for each country. The annual number of livebirths was calculated by dividing the TFR by 35 (the number of reproductive years based on the WHO definition) and multiplying the result by the number of women of reproductive age for each country and year. We extracted TFR and data for women of reproductive age from the WHO Global Health Observatory (GHO) and used mid-year resident numbers. The GHO derived these data from the World Population Division of the UN. When multiple data sources were available from the UN, we used civil registration systems and population censuses as preferential sources. If these data were not available, we used household surveys.

We extracted country-specific data on stillbirth numbers from UNICEF from 2000 to 2019, based on estimates from the UN Core Stillbirth Estimation Group (appendix pp 1–3).^18^ As 2020 data on stillbirths were not available at the time of this study, we used 2019 data for 2020 values.

WHO region-specific data on induced abortion rates per 1000 women of reproductive age were provided by Jonathan Bearak (Guttmacher Institute, New York, NY, USA; based on data from their group publications^19^, ^20^, ^21^). We calculated the number of induced abortions in each country by dividing the number of women of reproductive age for a given country by 1000 and multiplying the result by the inducted abortion rate of the respective WHO regions. As for stillbirths, 2019 rates for induced abortions were used for 2020 values (appendix p 1).

Methods for imputation of data for countries with missing demographic data are described in the appendix (pp 1–2).

Country-specific data on miscarriages are not available. Therefore, the same method was applied as described previously^10^ to determine the proportion of pregnancies resulting in miscarriage (appendix p 3).

The estimates of pregnancies at risk of malaria were determined by multiplying the total number of pregnancies each year by the proportion of the population living in areas with malaria transmission. The estimates of pregnancies at risk of malaria was also done by pregnancy outcomes, endemicity strata, and parasite species. 95% UIs for the estimates of pregnancies occurring in areas of *P falciparum* and *P vivax* malaria transmission by endemicity strata were derived from UIs for the population at risk using the same calculation as for the overall risk. Analyses were done in R (versions 4.1.2) and SPSS (version 26 or later).

### Role of the funding source

The funders of the study had no role in study design, data collection, data analysis, data interpretation, or writing of the report.

## Results

In 2020, there were 247·7 million pregnancies worldwide, of which 156·9 million (63·4%) were in 85 countries where malaria was endemic (table 1; appendix pp 4–5). Of these 247·7 million pregnancies, 121·9 million occurred within the spatial limits of *P falciparum* or *P vivax* malaria transmission and were therefore at risk of malaria, representing 77·7% of all pregnancies in malaria-endemic countries and 49·2% worldwide. An estimated 70·9 million (58·1%) of these 121**·**9 million pregnancies at risk of malaria were estimated to have resulted in livebirths. We estimated the remainder to have resulted in pregnancy loss, with 1**·**4 million (1·1%) ending in stillbirths, 33·5 million (27·5%) in induced abortions, and 16·1 million (13·2%) in miscarriages. There were 67·7 million pregnancies at risk of both *P falciparum* and *P vivax* malaria transmission (table 1).

**Table 1:** Estimates of pregnancies (in millions) worldwide, in malaria-endemic countries and within areas of *Plasmodium falciparum* and *Plasmodium vivax* malaria transmission by pregnancy outcomes and WHO regions in 2020

	**All areas**		**Areas with either *P falciparum* or P *vivax malaria* transmission**					**Areas with both *P falciparum* and P *vivax malaria* transmission**				
	All pregnancies	Pregnancies in malaria-endemic countries*	Pregnancies at risk of malaria†	Livebirths at risk of malaria‡	Pregnancies at risk of malaria ending in stillbirths‡	Pregnancies at risk of malaria ending in induced abortions‡	Pregnancies at risk of malaria ending in miscarriage‡	Pregnancies at risk of malaria†	Livebirths at risk of malaria§	Pregnancies at risk of malaria§	Pregnancies at risk of malaria ending in induced abortions§	Pregnancies at risk of malaria ending in miscarriage§
AFRO	51·6	49·9 (96·7%)	46·1 (37·8%)	31·2 (67·7%)	0·8 (1·6%)	7·9 (17·1%)	6·3 (13·6%)	5·3 (7·9%)	3·5 (65·6%)	0·1 (1·7%)	1·0 (19·4%)	0·7 (13·4%)
AMRO	25·9	15·3 (58·9%)	6·7 (5·5%)	3·7 (55·3%)	0·0 (0·5%)	2·0 (30·6%)	0·9 (13·6%)	3·3 (4·9%)	1·8 (54·3%)	0·0 (0·5%)	1·1 (31·7%)	0·4 (13·6%)
EMRO	30·1	20·2 (67·1%)	11·1 (9·1%)	6·3 (57·1%)	0·2 (1·6%)	3·2 (28·6%)	1·4 (12·7%)	9·9 (14·5%)	5·7 (57·8%)	0·2 (1·7%)	2·7 (27·3%)	1·2 (12·7%)
EURO	23·3	0·0	0·0	0·0	0·0	0·0	0·0	0·0	0·0	0·0	0·0	0·0
SEARO	63·7	61·3 (96·2%)	52·9 (43·4%)	27·1 (51·3%)	0·4 (0·8%)	18·5 (34·9%)	6·9 (13·0%)	45·9 (67·7%)	23·6 (51·5%)	0·4 (0·8%)	15·9 (34·7%)	6·0 (13·0%)
WPRO	53·0	10·3 (19·4%)	5·1 (4·2%)	2·5 (48·4%)	0·0 (0·5%)	2·0 (38·2%)	0·7 (13·0%)	3·4 (5·0%)	1·7 (49·0%)	0·0 (0·5%)	1·3 (37·4%)	0·4 (13·0%)
Global	247·7	156·9 (63·4%)	121·9 (100·0%)	70·9 (58·1%)	1·4 (1·1%)	33·5 (27·5%)	16·1 (13·2%)	67·7 (100·0%)	36·3 (53·6%)	0·6 (0·9%)	22·0 (32·5%)	8·8 (13·0%)

Data are in n (millions; %) unless specified. AFRO=Regional Office for Africa. AMRO=Regional Office for the Americas. EMRO=Eastern Mediterranean Regional Office. EURO=Regional Office for Europe. SEARO=Regional Office for South-East Asia. WPRO=Regional Office for the Western Pacific.

* Denominator is all pregnancies in that region.

† Denominator is the global number of pregnancies at risk of malaria denoted in the last row of the column.

‡ Denominator is pregnancies at risk of malaria in areas with either *P falciparum* or *P vivax* malaria transmission by region.

§ Denominator is pregnancies at risk of malaria in areas with both *P falciparum* and *P vivax* malaria transmission by region.

**Figure 1:**
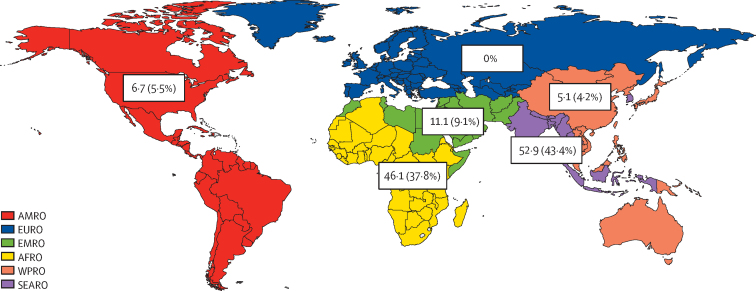
Distribution of pregnancies occurring in areas of *Plasmodium falciparum* and *Plasmodium vivax* malaria transmission in 2020 by WHO regions (proportion of global estimates) Data are in millions. AFRO=Regional Office for Africa. AMRO=Regional Office for the Americas. EMRO=Eastern Mediterranean Regional Office. EURO=Regional Office for Europe. SEARO=Regional Office for South-East Asia. WPRO=Regional Office for the Western Pacific.

Of the 121·9 million pregnancies at risk of malaria, 52·9 million (43·4%) occurred in countries in the WHO regional offices for South-East Asia (SEARO), 5·1 million (4·2%) in the Western Pacific (WPRO) region, 46·1 million (37·8%) in the Africa (AFRO) region, 11·1 million (9·1%) in the Eastern Mediterranean (EMRO) region, and 6**·**7 million (5·5%) in the Americas (AMRO; figure 1) region. The data by country are shown in the appendix (pp 6–11). Overall, there were 110·8 million pregnancies at risk of *P falciparum* and 78·7 million at risk of *P vivax* in 2020 (table 2). The sensitivity analysis using the alternative MAP model (ie, the whole of sub-Saharan Africa is considered endemic for *P vivax*) to estimate populations at risk suggests that estimates for the population at risk of *P vivax* would be seven-times higher (36·6 million) for AFRO than in the main analysis.

**Table 2:** Estimates of pregnancies (in millions) occurring in areas of malaria transmission by species, region, and endemicity in 2020

	**Plasmodium falciparum**					**Plasmodium vivax**	
	Hypoendemic areas	Mesoendemic areas	Hyperendemic areas	Holoendemic areas	Overall*, n (%)†	Hypoendemic areas	Overall*, n (%)†
AFRO	16·77 (10·87–24·67)	25·01 (17·31–32·09)	3·14 (0·84–7·55)	0·51 (0·16–2·45)	46·11 (41·6%)	5·33 (5·33–5·33)	5·33 (6·8%)
AMRO	4·51 (4·4–4·53)	0·00 (0·00–0·02)	0·0 (0·00–0·00)	0	4·52 (4·1%)	5·42 (5·10–5·42)	5·42 (6·9%)
EMRO	10·03 (8·44–10·37)	0·41 (0·08–1·13)	0·00 (0·00–0·03)	0	10·46 (9·4%)	10·43 (10·43–10·43)	10·43 (13·2%)
EURO	0	0	0	0	0	0	0
SEARO	45·96 (45·92–45·97)	0·01 (0·00–0·04)	0	0	45·97 (41·5%)	52·77 (52·77–52·77)	52·77 (67·0%)
WPRO	3·74 (3·71–3·75)	0·01 (0·00–0·04)	0	0	3·76 (3·4%)	4·75 (4·75–4·75)	4·75 (6·0%)
Global‡	81·04 (73·35–89·32)	25·47 (17·41–33·35)	3·15 (0·84–7·59)	0·52 (0·16–2·45)	110·84 (100·0%)	78·72 (78·40–78·72)	78·72 (100·0%)

Data are n (millions; 95% uncertainty interval) unless specified. The definition of endemicity strata was based on the parasite point prevalence in individuals aged 2–10 years for *P falciparum* and 1–99 years for *P vivax*. 0–10% was considered hypoendemic, >10–50% mesoendemic; >50–75% hyperendemic, and >75% holoendemic. AFRO=Regional Office for Africa. AMRO=Regional Office for the Americas. EMRO=Eastern Mediterranean Regional Office. EURO=Regional Office for Europe. SEARO=Regional Office for South-East Asia. WPRO=Regional Office for the Western Pacific.

* Different methods have been used by MAP to derive overall estimates and estimates by endemicity strata; as a result, the sum of the endemicity strata is not always equivalent to the overall estimate.

† Denominator is the global number of pregnancies at risk of malaria by species denoted in the final row of the column.

‡ Due to rounding and some estimates in individual cells being less than 10 000, the total might be different to the other estimates presented.

81·0 million (73·6%) of 110.8 million pregnancies at risk of *P falciparum* in 2020 occurred in hypoendemic transmission areas, 25·5 (23·1%) in mesoendemic transmission areas, 3·2 (2·9%) in hyperendemic transmission areas, and 0·5 (0·5%) in holoendemic transmission areas (table 2). *P vivax* transmission occurred exclusively in areas of hypoendemic transmission.

**Figure 2:**
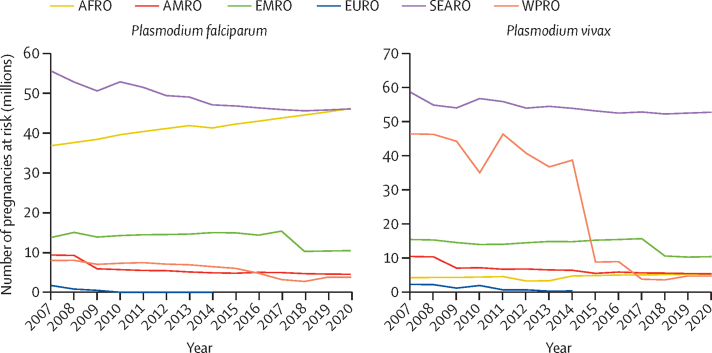
Number of pregnancies occurring in areas of *Plasmodium falciparum* and *Plasmodium vivax* transmission in 2007–20, by WHO regions AFRO=Regional Office for Africa. AMRO=Regional Office for the Americas. EMRO=Eastern Mediterranean Regional Office. EURO=Regional Office for Europe. SEARO=Regional Office for South-East Asia. WPRO=Regional Office for the Western Pacific.

Comparison over time showed that, globally, the number of pregnancies at risk fell by 28·8%, from 171·2 million in 2007 to 121·9 million in 2020 (figure 2). Pregnancies in *P falciparum* transmission areas fell by 11·4%, from 125·1 million in 2007 to 110·8 million in 2020, and those in *P vivax* transmission areas fell by 42·8%, from 137·5 million in 2007 to 78·7 million in 2020. The reduction in pregnancies in *P falciparum* transmission areas was seen in all WHO regions except AFRO, where the number increased by 25·4% (from 36·8 million to 46·1 million, figure 2). The WHO Europe (EURO) region had a complete drop in pregnancies at risk of *P falciparum,* with the last *P falciparum* transmission reported in 2009. The second-largest decrease in pregnancies at risk of *P falciparum* transmission was in the WPRO region, where at-risk pregnancies fell by 52·6% (from 7·9 million in 2007 to 3·8 million in 2020). In the AMRO region, pregnancies at risk fell by 51·5% (from 9·3 million to 4·5 million) in AMRO, 23·9% (from 13·7 million to 10·5 million) in EMRO, and 17·2% (from 55·5 million to 46·0 million) in SEARO.

Similarly, pregnancies at risk in areas of *P vivax* transmission fell in all regions except AFRO, which saw a 25·8% increase (from 4·2 million in 2007 to 5·3 million in 2020). There has been no *P vivax* transmission in the EURO region since 2014. The second largest reduction, 89·8%, occurred in WPRO (46**·**4 million to 4**·**8 million), followed by 48·4% in AMRO (10**·**5 million to 5**·**4 million), 32·4% in EMRO (15·4 million to 10·4 million) and 10·0% in SEARO (58·6 million to 52·8 million). The largest reduction in pregnancies at risk of *P vivax* globally, 26·3%, occurred between 2014 (119·0 million) and 2015 (87·7 million).

**Figure 3:**
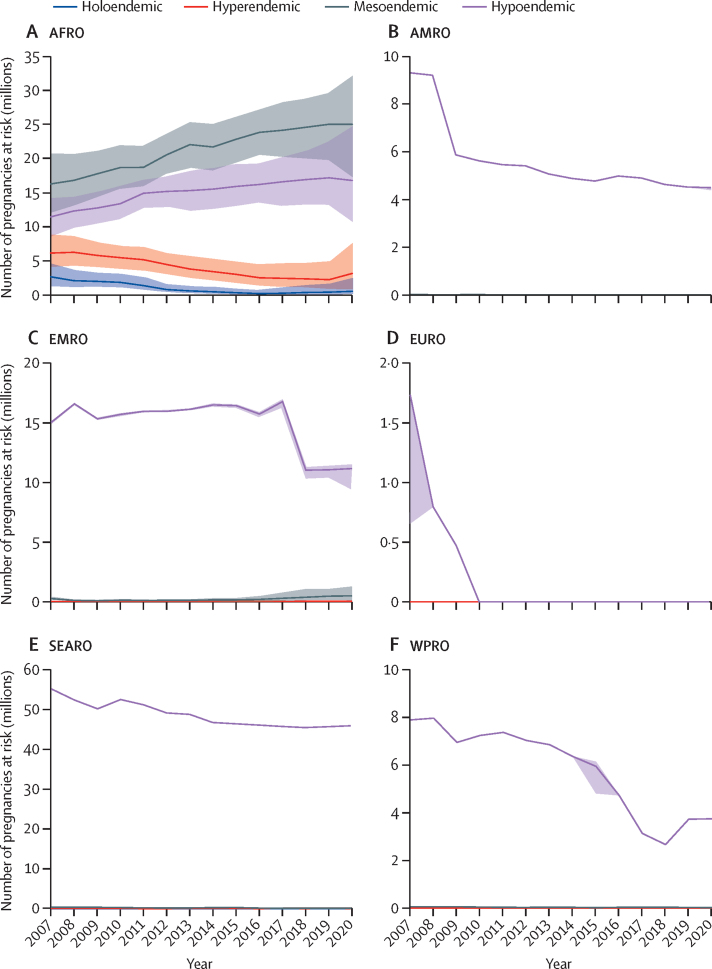
Estimates of pregnancies in areas of *Plasmodium falciparum* transmission in 2007–20 by endemicity and by WHO region with 95% uncertainty intervals The definition of endemicity strata is based on the parasite point prevalence in individuals aged 2–10 years for *P falciparum* and in individuals aged 1–99 years for *P vivax*. 0–10% was considered hypoendemic, >10–50% mesoendemic, >50–75% hyperendemic, and >75% holoendemic. AFRO=Regional Office for Africa. AMRO=Regional Office for the Americas. EMRO=Eastern Mediterranean Regional Office. SEARO=Regional Office for South-East Asia. WPRO=Regional Office for the Western Pacific.

Further stratification of time trends by malaria endemicity in the AFRO region showed that the number of pregnancies at risk of *P falciparum* transmission reduced in holoendemic settings (80·4%, from 2·7 million in 2007 to 0·5 million in 2020) and hyperendemic (48·7%, from 6·1 million to 3·1 million), whereas the number at risk increased in mesoendemic (53·7%, from 16·3 million to 25·0 million) and hypoendemic areas (46·7%, from 11·4 million to 16·8 million; figure 3). In the remaining regions, *P falciparum* transmission occurred primarily in mesoendemic and hypoendemic areas. AMRO had a reduction in pregnancies at risk, primarily driven by a 51·5% decline in the number of pregnancies at risk in hypoendemic transmission areas (from 9·3 million in 2007 to 4·5 million in 2020). The number of pregnancies at risk in areas of mesoendemic transmission also declined by 54·2% (from 0·014 million to 0·007 million). In the EMRO region, pregnancies at risk of *P falciparum* infection increased from 0 to 0·002 million for hyperendemic areas, doubled (from 0·2 million to 0·4 million) in mesoendemic areas, and decreased by 25·9% (from 13·5 million to 10·0 million) in hypoendemic areas. In the EURO region, *P falciparum* transmission declined to 0 in 2010. In the SEARO region, the number of pregnancies at risk in hypoendemic settings reduced by 16·7%, from 55·2 million in 2007 to 46·0 million in 2020. Those in mesoendemic settings reduced by 84·6%, from 0·34 million to 0·01 million. In the WPRO region, the number of pregnancies in hypoendemic transmission areas reduced by 52·5%, from 7·9 million to 3·7 million and in mesoendemic areas by 63·7%, from 0·05 million to 0·02 million (figure 3).

## Discussion

We estimate that 121·9 million pregnancies occurred in malaria transmission areas in 2020, resulting in 70·9 million livebirths. Although sub-Saharan Africa has the most malaria-associated adverse pregnancy outcomes, it represents only 37·8% of pregnancies at risk of *P falciparum* or *P vivax*. Just under half of the pregnancies at risk of malaria occurred in SEARO and WPRO. Globally, the number of pregnancies occurring in areas with malaria transmission decreased by 28·8% from 2007 to 2020, despite a 7·0% increase in the annual number of pregnancies from 231·5 million to 247·7 million. The biggest reduction (42·8%) occurred in areas of *P vivax* transmission (from 137·5 million to 78·7 million), compared with only 11·4% for *P falciparum* (from 125·1 million to 110·8 million).

Pregnancies at risk of *P falciparum* or *P vivax* in the AFRO region increased by 25·4% between 2007 and 2020. However, considering the 28·2% increase in the number of pregnancies in AFRO in the same period, the data reflect positive movement towards shrinking the malaria transmission map in this region. For *P falciparum*, stratification of time trends by malaria endemicity showed that pregnancies at risk fell in holoendemic (by 80·4%) and hyperendemic (by 48·7%) settings and increased in mesoendemic (by 53·7%) and hypoendemic (by 46·7%) areas. These data correspond to about 5·1 million fewer pregnancies in people living in the highest transmission areas (holoendemic and hyperendemic) in these 14 years between 2007 and 2020. This change reflects a region-wide decline in *P falciparum* transmission, as seen by the substantial declines in the case burden of malaria in the general population.^11^ *P vivax* transmission occurred only in the lowest endemicity strata over the study period. Our primary estimates for the number of pregnancies at risk of *P vivax* transmission in Africa were restricted to the horn of Africa. This restriction might represent a conservative estimate of the actual number of pregnancies at risk as this model's geospatial boundaries for *P vivax* transmission were derived from documented *P vivax* surveillance data, which are mostly based on microscopy and rapid diagnostic tests. However, many *P vivax* infections are not detectable by these standard diagnostic tools, which do not detect vascular or extravascular subpatent (ie, malaria infections below the detection threshold of standard diagnostic tests), or latent infections seated in inaccessible tissues.^15^, ^22^ Our sensitivity analysis suggests that the number of pregnancies at risk of *P vivax* in AFRO could be close to seven-times higher than reported in the main analysis, because, with this alternative method, all of sub-Saharan Africa is considered endemic for *P vivax*, not just the horn of Africa. Better diagnostic tools and data are needed to estimate the true global distribution of *P vivax* endemicity.

Our estimates for pregnancies at risk of *P falciparum* and *P vivax* that resulted in livebirths for the WHO AFRO region (31·2 million in 2020) are consistent with the latest estimates by WHO for 2020 (33·8 million).^8^ Our work improves on the estimate by WHO, which is based on livebirths only and thus underestimates the total number of pregnancies at risk in Africa because only 68% of pregnancies were estimated to result in livebirths in this region in 2020. The total number of pregnancies at risk is more informative for policy makers as malaria in pregnancy is a major cause of miscarriage and stillbirths.^1^, ^23^, ^24^

Our current estimate of the pregnancies at risk of *P falciparum* or *P vivax* (171·2 million) in 2007 is 36·7% higher than our previous estimate for 2007 (125·2 million).^10^ The differences are driven by the 21·4% increase in the risk estimate for the overall population at risk in the latest MAP model compared with older models.^25^, ^26^ The latest MAP models provide improved estimates by incorporating routine surveillance data, increasing the information available for areas outside of AFRO. In addition, estimates for induced abortion were 59·9% higher following the improved knowledge of induced abortion numbers, particularly in informal settings.^19^

The strengths of this study include the use of the most up-to-date estimates of the global spatial limits of malaria transmission by species and endemicity strata from MAP. Furthermore, the multiple data sources for livebirth, miscarriage, stillbirths, and induced abortion estimates were cross-referenced with alternative data sources.

The study has several limitations that could affect the accuracy of our estimates. First, the data on the population living within the global spatial limits of malaria transmission is affected by the same survey and routine surveillance data limitations reported previously.^11^ Second, our results depend on the accuracy of the modelled prevalence and incidence surfaces generated by MAP. Furthermore, as MAP contributes to both the World Malaria Report and the Global Burden of Diseases reports, cross-referencing with alternate suitable data sources for malaria is challenging. Nevertheless, the methods used by MAP are now widely accepted in the malaria research community.^12^ A sensitivity analysis using a variation of the primary model to estimate the population at risk of malaria indicated that our results of pregnancies at risk of *P vivax* transmission in sub-Saharan Africa could be underestimated. In the future, estimates based on advanced diagnostics might be able to account for subpatent infection, improving determination of geospatial distribution and pregnancies at risk of *P vivax* infections.^15^ Third, our pregnancy estimates were limited by potential errors and imprecisions in TFR and pregnancy outcomes, including stillbirth and induced abortion, as well as the derived estimates for miscarriage rate. Fertility rate estimates were limited by uncertainties about maternal age and under-reporting of livebirths for neonates that die before they are registered. Therefore, low coverage of birth and stillbirth registration data by civil registration systems and under-reporting in censuses and household surveys could lead to inaccurate estimates.^27^ Furthermore, data for induced abortions were available by WHO regions rather than countries, which could have concealed variations between countries and lead to overestimation or underestimation of induced abortion rates. It was therefore reassuring to cross-check the rates of stillbirth, livebirths, and induced abortions with alternative data sources. Overall, there is a dearth of data on miscarriage, and our estimates use a blanket formula applied for all countries, which might miss differences in risk of miscarriage across settings. Fourth, some estimates of the number of women of reproductive age and pregnancy-related demographic data were unavailable for 44 countries, which had to be imputed from neighbouring countries with similar demographics and could have introduced some inaccuracies. The absence of pregnancy-related demographic data affected only two malaria-endemic countries, French Guiana and Taiwan, which should have a limited effect on our estimates for pregnancies at risk of malaria. Finally, it is important to emphasise that the data presented are our best approximations of pregnancies at risk of malaria. Although UIs were available for our estimates by endemicity strata, we were unable to provide similar UIs for models of the overall number of pregnancies at risk of *P falciparum* and *P vivax* malaria or to incorporate uncertainty for some pregnancy covariates, such as TFR and miscarriage, which have their own uncertainties.

Our study provides the most up-to-date contemporary estimates and trend in estimates of pregnancies at risk of malaria by parasite species and endemicity at regional and global levels. In this study, we also provide an informed platform to estimate the disease and economic burden of malaria in pregnancy and its spatial distribution and to monitor the effect of malaria control efforts. Our findings might guide priority setting for resource allocation for the control of malaria in pregnancy to areas of greatest need.

## Data sharing

All aggregated country-level data collected during this analysis will be made available for a period of 5 years after publication. Access to data will be provided following the investigators’ approval of a proposal after considering the overlap between the proposal and any ongoing efforts. Proposals should be directed to the corresponding authors. People requesting data will need to sign a data access agreement and the database will be transferred electronically. The study protocol will be made available on request. For raw data: pixel-level and administrative-level summaries of populations at risk of malaria are available for visualisation and download at https://malariaatlas.org/data-directory/; number of women of reproductive age (country level) and total fertility rates (country level) at https://www.who.int/data/gho; stillbirth rates (country level) at https://data.unicef.org/dv_index/; and induced abortion rates (country level) at https://osf.io/5k7fp/.

## Declaration of interests

We declare no competing interests.
