# Supplementary material for: Global estimates of the number of pregnancies at risk of malaria from 2007 to 2020: a demographic study
Source: Lancet Glob Health. 2022 Dec 13;11(1):e40–7. doi: 10.1016/S2214-109X(22)00431-4 (PMC9764451; doi:10.1016/S2214-109X(22)00431-4)
Supplement: Supplementary appendix [file mmc1.pdf]

THE LANCET
Global Health

Supplementary appendix

This appendix formed part of the original submission and has been peer reviewed. We post it as supplied by the authors.

Supplement to: Reddy V, Weiss DJ, Rozier J, ter Kuile FO, Dellicour S. Global estimates of the number of pregnancies at risk of malaria from 2007 to 2020: a demographic study. *Lancet Glob Health* 2023; **11:** e40–47.

Supplementary appendix

Supplement to: Reddy et al., Global estimates of the number of pregnancies at risk of malaria from 2007 to 2020: a demographic study

Supplemental methods

Data sources

Annual number of pregnancies per country

Live births and women of reproductive age for countries with missing data

Data on women of reproductive age (WORA) numbers and total fertility rate (TFR) were extracted from United Nations Population Data Portal[1] for 42 countries not covered from the WHO Global Health Observatory (GHO). For 23 countries without data available from the UN, the TFR and proportion of the total population that were WORAs were imputed from neighbouring countries. For example, Suriname was used to calculate the proportion of the population that are WORAs in French Guiana. The number of WORAs in these two countries was then calculated as (the total population x the % of WORAs in the country with similar demographics).

Stillbirths

Country-specific data on stillbirth numbers were extracted from UNICEF for the years 2000 to 2019, based on estimates from the UN Core Stillbirth Estimation Group (CSEG).[2] As 2020 data on stillbirths were not available at the time of this study, 2019 values were used for 2020. The CSEG uses a hierarchical Bayesian model to produce nationally representative stillbirth estimates for each UN country using the WHO definition of stillbirth ("a baby who dies after 28 weeks of pregnancy, but before or during birth"). They input data from four sources: administrative systems, including vital registration systems, health management information systems, household surveys, and population studies. Factors correlated with stillbirths, such as demographic and biomedical factors, are used to account for the lack of reporting of stillbirth data.[3]

Induced abortions

Country-specific data on induced abortion rates per 1000 WORAs for 2015-2019 was obtained from a published review by Bearak et al., 2020.[4] Upon request, the authors also shared data by WHO region for the years 2007-2019, provided in 5-yearly intervals (2005-2019, 2010-2014 and 2015-2019) as published by Popinchalk et al. in 2021.[5]

Popinchalk et al. calculated induced abortion rates using a Bayesian framework, applied to data from regional surveys; official statistics; published reviews obtained through literature search; and data from World Population Prospects on live births.[5] The number of induced abortions in each country was then calculated by dividing the number of WORAs for a given country by 1000 and multiplying the result by the inducted abortion rate of the respective WHO region. As was the case for stillbirths, 2019 rates for induced abortions were used for 2020.

Stillbirth and Induced abortions for countries with missing data

For countries where stillbirth and induced abortion data were not available, stillbirth and abortion rates were derived from a matched country selected based on proximity and fertility rate. The list of countries with imputed stillbirth and abortion data is provided in table S1 below.

Table S1. Countries with missing stillbirth or abortion data and those matched for imputations

Country with missing data	Matched country to impute data
Andorra	France
Anguilla	Antigua
Aruba	Grenada
Bermuda	Antigua
Cayman Islands	Jamaica
Christmas Island	Indonesia
Cocos (Keeling) Islands	Indonesia
Dhekelia and Akrotiri SBA	Cyprus
Dominica	Antigua
Falkland Islands (Malvinas)	Argentina
French Guiana	Suriname
Gibraltar	Spain
Guadeloupe	Antigua
Guam	Micronesia
Guernsey	UK
Holy See (Vatican City State)	Italy
Hong Kong	Singapore
Jersey	UK
Liechtenstein	Switzerland
Macau	Singapore
Marshall Islands	Micronesia
Martinique	Antigua
Mayotte	Comorros
Monaco	France
Montserrat	Antigua
Nauru	Micronesia
Netherlands Antilles	Grenada
New Caledonia	Vanuatu
Norfolk Island	Vanuatu
Northern Mariana Islands	Micronesia
Palau	Micronesia
Palestine	Jordan
Paracel Islands	Philipines
Puerto Rico	Jamaica
Reunion	Mauritius
Saint Helena	Angola
Saint Kitts and Nevis	Antigua
Saint Pierre and Miquelon	Canada
San Marino	Italy
Taiwan	Japan
Turks and Caicos Islands	Dominican Republic
Virgin Islands (US)	Antigua
Virgin Islands, British	Antigua
Western Sahara	Morocco

Miscarriage

Country-specific data on miscarriages are not available. Therefore, the same method was applied as described previously in our original paper to determine the proportion of pregnancies resulting in miscarriage.[6] In brief, the method uses multipliers to work backwards from the known number live births and induced abortions to recover the unknown underlying number of pregnancies that 'produced' them, as described elsewhere.[7-10] It assumes that the number of spontaneous pregnancy losses (stillbirths and miscarriages) is 10% of induced abortions plus 20% of live births.[9,10] The stillbirth rate obtained from UNICEF was then subtracted from the obtained spontaneous pregnancy loss for each country to deduce miscarriage rates.[10,11]

References

1. Nations U. UN Population Data Portal. https://population.un.org/dataportal/home (accessed Aug 2022.
2. UNICEF. Stillbirth Estimates: Country Specific Stillbirth Estimates (numbers). 2020 ed. UNICEF GLOBAL DATABASES: UNICEF GLOBAL DATABASES, UN IGME and its UN Core Stillbirth Estimation Group; 2020.
3. World Health Organization. Making Every Baby Count - Audit and review of stillbirths and neonatal deaths. *World Health Organization, Geneva* 2016.
4. Bearak J, Popinchalk A, Ganatra B, Moller A-B, Tuncalp O, Beavin C. Unintended pregnancy and abortion by income, region and the legal status of abortion: estimated from a comprehensive model 1990-2019. *The Lancet, Global Health* 2020; **8**(9).
5. Popinchalk A, Beavin C, Bearak J. The state of global abortion data: an overview and call to action. *BMJ Sex Reprod Health* 2022; **48**(1): 3-6.
6. Dellicour S, Tatem AJ, Guerra CA, Snow RW, ter Kuile FO. Quantifying the Number of Pregnancies at Risk of Malaria in 2007: A Demographic Study. *PLOS Medicine* 2010; **7**(1).
7. Sedgh G, Henshaw S, Singh S, Ahman E, Shah IH. Induced abortion: estimated rates and trends worldwide. *Lancet* 2007; **370**(9595): 1338-45.
8. Leridon. H. Human Fertility: The Basic Components (Table 4.20). Chicago: University of Chicago Press; 1977.
9. Hammerslough CR. Estimating the probability of spontaneous abortion in the presence of induced abortion and vice versa. *Public Health Rep* 1992; **107**(3): 269-77.
10. Henshaw SK, Binkin NJ, Blaine E, Smith JC. A portrait of American women who obtain abortions. *Fam Plann Perspect* 1985; **17**(2): 90-6.
11. UNICEF. Stillbirths estimates, notes on the data. 2020. https://data.unicef.org/topic/child-survival/stillbirths/ (accessed 23/08/2021 2021).

Supplemental tables

Table S2: Estimates of pregnancies occurring in malaria-endemic countries and within areas of malaria transmission by pregnancy outcome, WHO region and countries in 2020 (in 1000s).

WHO Region	Country	Pregnancies	Pregnancies in areas of Pf or Pv malaria transmission	Pregnancy Outcomes – in areas of *Pf* or *Pv* malaria transmission			
				Live Births	Stillbirths	Induced Abortions	Miscarriages
AFRO	Angola	1,686	1,641	1,137	25	252	227
AFRO	Benin	572	572	387	9	98	78
AFRO	Botswana	86	42	25	0	11	6
AFRO	Burkina Faso	1,026	1,026	703	15	166	142
AFRO	Burundi	606	551	380	11	86	74
AFRO	Cameroon	1,226	1,222	818	18	219	168
AFRO	Cape Verde	17	2	1	0	0	0
AFRO	Central African Republic	221	221	149	5	39	29
AFRO	Chad	852	813	566	18	121	107
AFRO	Comoros	38	38	25	1	7	5
AFRO	Congo (Brazzaville)	247	246	164	3	45	35
AFRO	Congo, (Kinshasa)	4,679	4,527	3,168	96	659	604
AFRO	Côte d'Ivoire	1,238	1,238	832	22	218	166
AFRO	Equatorial Guinea	58	56	38	1	10	8
AFRO	Eritrea	145	136	89	2	27	19
AFRO	Ethiopia	5,025	4,049	2,658	73	781	537
AFRO	Gabon	94	94	61	1	19	13
AFRO	Gambia	123	123	85	2	20	17
AFRO	Ghana	1,286	1,286	831	20	263	173
AFRO	Guinea	623	623	419	12	109	83
AFRO	Guinea-Bissau	91	90	60	2	17	12
AFRO	Kenya	2,147	1,767	1,112	25	393	237
AFRO	Liberia	221	220	146	4	41	29
AFRO	Madagascar	1,190	1,149	751	14	225	159
AFRO	Malawi	831	831	545	10	160	115
AFRO	Mali	1,056	1,037	725	16	152	144
AFRO	Mauritania	216	189	126	3	34	26
AFRO	Mozambique	1,504	1,503	1,017	25	257	204
AFRO	Namibia	101	62	39	1	14	9
AFRO	Niger	1,368	1,335	957	21	169	188
AFRO	Nigeria	10,336	10,335	7,131	171	1,617	1,416
AFRO	Rwanda	568	463	302	6	92	64
AFRO	Sao Tome and Principe	9	9	6	0	2	1
AFRO	Senegal	788	787	528	11	140	108
AFRO	Sierra Leone	348	348	229	6	67	46
AFRO	South Africa	1,903	219	125	2	63	29
AFRO	South Sudan	520	520	349	12	91	67
AFRO	Swaziland	43	1	1	0	0	0
AFRO	Tanzania	2,837	2,793	1,894	40	472	386
AFRO	Togo	370	370	245	6	69	50
AFRO	Uganda	2,173	2,118	1,433	29	363	294
AFRO	Zambia	863	863	579	10	153	122
AFRO	Zimbabwe	602	598	379	7	130	82
AMRO	Bolivia	378	239	143	1	61	33

WHO Region	Country	Pregnancies	Pregnancies in areas of Pf or Pv malaria transmission	Pregnancy Outcomes – in areas of *Pf* or *Pv* malaria transmission			
				Live Births	Stillbirths	Induced Abortions	Miscarriages
AMRO	Brazil	5,359	2,146	1,116	9	733	288
AMRO	Colombia	1,319	781	412	3	261	105
AMRO	Costa Rica	124	122	63	0	41	17
AMRO	Dominican Republic	321	169	97	1	47	23
AMRO	Ecuador	539	346	201	2	95	48
AMRO	French Guiana	11	11	7	0	2	2
AMRO	Guatemala	634	197	120	2	48	27
AMRO	Guyana	24	24	14	0	6	3
AMRO	Haiti	406	373	228	5	90	50
AMRO	Honduras	319	189	110	1	51	26
AMRO	Mexico	3,714	644	359	3	194	89
AMRO	Nicaragua	209	115	67	1	32	16
AMRO	Panama	129	16	9	0	4	2
AMRO	Peru	969	711	405	3	205	98
AMRO	Suriname	17	17	10	0	5	2
AMRO	Venezuela	804	591	337	4	169	81
EMRO	Afghanistan	1,842	1,645	985	32	421	207
EMRO	Djibouti	38	7	3	0	2	1
EMRO	Iran	2,985	1,351	638	5	532	176
EMRO	Oman	149	53	28	0	18	7
EMRO	Pakistan	9,554	3,914	2,201	78	1,158	478
EMRO	Saudi Arabia	1,136	200	96	1	77	26
EMRO	Somalia	922	908	592	17	180	119
EMRO	Sudan	2,176	2,021	1,219	29	507	265
EMRO	Yemen	1,372	952	546	15	270	121
SEARO	Bangladesh	5,284	1,191	591	16	438	146
SEARO	Bhutan	23	3	1	0	1	0
SEARO	India	43,275	41,731	21,507	328	14,476	5,420
SEARO	Indonesia	8,923	6,851	3,580	35	2,323	913
SEARO	Myanmar	1,794	1,792	913	13	633	232
SEARO	Nepal	988	578	278	6	222	72
SEARO	South Korea	978	734	272	0	370	91
WPRO	Cambodia	622	620	315	5	220	80
WPRO	Laos	278	278	144	3	96	36
WPRO	Malaysia	1,056	1,056	489	3	426	137
WPRO	North Korea	769	757	344	3	313	97
WPRO	Papua New Guinea	392	343	197	3	97	46
WPRO	Philippines	3,964	196	100	1	69	26
WPRO	Solomon Islands	33	32	20	0	8	5
WPRO	Vanuatu	14	13	8	0	4	2
WPRO	Vietnam	3,157	1,847	870	7	729	240

Acronyms: AFRO=Regional Office for Africa, AMRO=Regional Office for the Americas, EMRO=Eastern Mediterranean Regional Office, Pf=P. falciparum, Pv=P. vivax, SEARO=Regional Office for South-East Asia, WPRO=Regional Office for the Western Pacific.

Table S3: Estimate of pregnancies occurring in areas of malaria transmission by species, WHO region and endemicity in 2020 (in thousands).

WHO Region	Country	*P. falciparum*													*P. vivax*			
		Hypoendemic*	Lower UI	Upper UI	Mesoendemic*	Lower UI	Upper UI	Hyperendemic*	Lower UI	Upper UI	Holoendemic*	Lower UI	Upper UI	Overall	Hypoendemic*	Lower UI	Upper UI	Overall
AFRO	Angola	350	181	1,009	1,091	465	1,293	115	59	266	51	20	125	1,641	-	-	-	-
AFRO	Benin	1	-	40	483	347	556	79	8	200	1	-	46	572	-	-	-	-
AFRO	Botswana	42	42	42	-	-	-	-	-	-	-	-	-	42	-	-	-	-
AFRO	Burkina Faso	19	-	349	729	432	955	175	15	444	16	-	209	1,026	-	-	-	-
AFRO	Burundi	1	-	90	457	347	549	75	-	185	-	-	7	551	-	-	-	-
AFRO	Cameroon	177	22	595	980	586	1,136	50	10	138	5	0	38	1,222	-	-	-	-
AFRO	Cape Verde	2	2	2	-	-	-	-	-	-	-	-	-	2	-	-	-	-
AFRO	Central African Republic	20	0	120	147	73	191	31	0	107	2	-	73	221	-	-	-	-
AFRO	Chad	350	122	615	440	198	641	5	-	70	-	-	13	813	-	-	-	-
AFRO	Comoros	38	-	38	-	-	38	-	-	-	-	-	-	38	-	-	-	-
AFRO	Congo (Brazzaville)	79	7	151	144	77	216	14	1	42	0	-	20	246	-	-	-	-
AFRO	Congo, (Kinshasa)	631	200	1,085	2,604	1,948	3,073	938	530	1,307	346	140	773	4,527	-	-	-	-
AFRO	Côte d'Ivoire	275	5	624	796	501	1,068	143	9	354	20	-	292	1,238	-	-	-	-
AFRO	Equatorial Guinea	1	-	33	53	23	56	0	-	17	-	-	-	56	-	-	-	-
AFRO	Eritrea	136	135	136	-	-	1	-	-	-	-	-	-	136	136	136	136	136
AFRO	Ethiopia					-		-	-	-	-	-						

**WHO Region**	**Country**	***P. falciparum***													***P. vivax***			
		**Hypoendemic***	**Lower UI**	**Upper UI**	**Mesoendemic***	**Lower UI**	**Upper UI**	**Hyperendemic***	**Lower UI**	**Upper UI**	**Holoendemic***	**Lower UI**	**Upper UI**	**Overall**	**Hypoendemic***	**Lower UI**	**Upper UI**	**Overall**
		4,035	3,879	4,049	14		170						-	4,049	4,048	4,048	4,048	4,048
AFRO	Gabon	25	2	68	60	24	84	3	0	29	0	-	9	94	-	-	-	-
AFRO	Gambia	123	123	123	-	-	0	-	-	-	-	-	-	123	-	-	-	-
AFRO	Ghana	368	130	554	905	720	1,148	11	0	53	0	-	1	1,286	-	-	-	-
AFRO	Guinea	58	1	298	461	309	571	62	2	198	4	-	89	623	-	-	-	-
AFRO	Guinea-Bissau	87	34	90	4	0	57	0	-	0	-	-	0	90	-	-	-	-
AFRO	Kenya	1,639	1,471	1,757	128	10	296	0	-	4	-	-	0	1,767	-	-	-	-
AFRO	Liberia	1	-	88	136	33	213	49	2	143	10	0	78	220	-	-	-	-
AFRO	Madagascar	983	670	1,099	166	51	479	0	0	6	-	-	0	1,149	1,149	1,149	1,149	1,149
AFRO	Malawi	172	8	626	646	205	787	0	-	107	-	-	0	831	-	-	-	-
AFRO	Mali	216	85	490	764	497	890	34	2	169	0	-	14	1,037	-	-	-	-
AFRO	Mauritania	159	50	187	29	2	138	-	-	0	-	-	-	189	-	-	-	-
AFRO	Mozambique	208	39	471	1,104	880	1,310	158	43	320	17	0	150	1,503	-	-	-	-
AFRO	Namibia	40	-	62	1	-	47	-	-	0	-	-	-	62	-	-	-	-
AFRO	Niger	221	10	822	1,011	483	1,239	48	-	452	-	-	6	1,335	-	-	-	-
AFRO	Nigeria	778	129	2,022	8,501	7,203	9,642	914	122	2,211	10	-	222	10,335	-	-	-	-
AFRO	Rwanda	462	462	463	1	0	2	-	-	-	-	-	-	463	-	-	-	-

WHO Region	Country	***P. falciparum***													***P. vivax***			
		**Hypoendemic***	**Lower UI**	**Upper UI**	**Mesoendemic***	**Lower UI**	**Upper UI**	**Hyperendemic***	**Lower UI**	**Upper UI**	**Holoendemic***	**Lower UI**	**Upper UI**	**Overall**	**Hypoendemic***	**Lower UI**	**Upper UI**	**Overall**
AFRO	Sao Tome and Principe	9	9	9	0	-	1	-	-	-	-	-	-	9	-	-	-	-
AFRO	Senegal	778	461	787	9	-	326	-	-	0	-	-	-	787	-	-	-	-
AFRO	Sierra Leone	30	-	139	247	108	342	53	-	185	-	-	109	348	-	-	-	-
AFRO	South Africa	219	219	219	-	-	0	-	-	-	-	-	-	219	-	-	-	-
AFRO	South Sudan	108	25	305	325	186	412	54	5	186	9	-	56	520	-	-	-	-
AFRO	Swaziland	1	1	1	-	-	0	-	-	-	-	-	-	1	-	-	-	-
AFRO	Tanzania	2,116	1,503	2,540	675	253	1,288	1	-	33	-	-	0	2,793	-	-	-	-
AFRO	Togo	63	0	174	285	196	368	0	-	36	-	-	-	370	-	-	-	-
AFRO	Uganda	699	217	1,084	1,259	930	1,732	110	40	206	26	0	102	2,118	-	-	-	-
AFRO	Zambia	459	185	626	359	230	625	24	2	84	0	-	25	863	-	-	-	-
AFRO	Zimbabwe	594	448	598	3	-	150	-	-	0	-	-	-	598	-	-	-	-
AMRO	Bolivia	-	-	-	-	-	-	-	-	-	-	-	-	-	239	239	239	239
AMRO	Brazil	1,422	1,420	1,422	0	-	2	-	-	-	-	-	-	1,422	2,018	2,018	2,018	2,018
AMRO	Colombia	686	685	686	0	-	1	-	-	0	-	-	-	686	780	780	780	780
AMRO	Costa Rica	122	122	122	-	-	-	-	-	-	-	-	-	122	-	-	-	-
AMRO	Dominican Republic	169	169	169	-	-	0	-	-	-	-	-	-	169	-	-	-	-
AMRO	Ecuador					-		-	-	-	-	-						

**WHO Region**	**Country**	***P. falciparum***													***P. vivax***			
		**Hypoendemic***	**Lower UI**	**Upper UI**	**Mesoendemic***	**Lower UI**	**Upper UI**	**Hyperendemic***	**Lower UI**	**Upper UI**	**Holoendemic***	**Lower UI**	**Upper UI**	**Overall**	**Hypoendemic***	**Lower UI**	**Upper UI**	**Overall**
		226	226	226	0		0						-	226	341	341	341	341
AMRO	French Guiana	9	-	9	-	-	0	-	-	-	-	-	-	9	11	11	11	11
AMRO	Guatemala	-	-	-	-	-	-	-	-	-	-	-	-	-	197	197	197	197
AMRO	Guyana	23	21	24	0	-	2	-	-	0	-	-	-	24	24	24	24	24
AMRO	Haiti	373	369	373	-	-	4	-	-	-	-	-	-	373	-	-	-	-
AMRO	Honduras	124	124	124	-	-	-	-	-	-	-	-	-	124	189	-	189	189
AMRO	Mexico	-	-	-	-	-	-	-	-	-	-	-	-	-	644	644	644	644
AMRO	Nicaragua	92	-	92	-	-	0	-	-	-	-	-	-	92	115	-	115	115
AMRO	Panama	-	-	-	-	-	-	-	-	-	-	-	-	-	16	16	16	16
AMRO	Peru	693	689	693	0	-	4	-	-	0	-	-	-	693	244	244	244	244
AMRO	Suriname	-	-	10	-	-	-	-	-	-	-	-	-	-	17	-	17	17
AMRO	Venezuela	581	576	585	6	3	11	1	-	2	-	-	-	588	591	591	591	591
EMRO	Afghanistan	1,578	1,570	1,578	-	-	8	-	-	-	-	-	-	1,578	1,645	1,645	1,645	1,645
EMRO	Djibouti	6	0	7	-	-	7	-	-	7	-	-	-	7	7	7	7	7
EMRO	Iran	855	-	855	-	-	-	-	-	-	-	-	-	855	1,313	1,313	1,313	1,313
EMRO	Oman	53	53	53	-	-	-	-	-	-	-	-	-	53	53	53	53	53
EMRO	Pakistan	3,896	3,884	3,900	6	2	17	0	-	1	-	-	-	3,902	3,914	3,914	3,914	3,914

**WHO Region**	**Country**	***P. falciparum***													***P. vivax***			
		**Hypoendemic***	**Lower UI**	**Upper UI**	**Mesoendemic***	**Lower UI**	**Upper UI**	**Hyperendemic***	**Lower UI**	**Upper UI**	**Holoendemic***	**Lower UI**	**Upper UI**	**Overall**	**Hypoendemic***	**Lower UI**	**Upper UI**	**Overall**
EMRO	Saudi Arabia	200	200	200	-	-	-	-	-	-	-	-	-	200	-	-	-	-
EMRO	Somalia	748	529	860	161	49	363	-	-	1	-	-	-	908	908	908	908	908
EMRO	Sudan	1,841	1,400	1,988	176	33	616	-	-	14	-	-	1	2,021	2,021	2,021	2,021	2,021
EMRO	Yemen	860	809	938	76	2	128	1	-	10	-	-	-	940	575	575	575	575
SEARO	Bangladesh	1,191	1,190	1,191	-	-	1	-	-	-	-	-	-	1,191	1,116	1,116	1,116	1,116
SEARO	Bhutan	3	3	3	-	-	-	-	-	-	-	-	-	3	3	3	3	3
SEARO	India	36,575	36,554	36,582	7	1	28	-	-	-	-	-	-	36,583	41,710	41,710	41,710	41,710
SEARO	Indonesia	5,989	5,975	5,993	4	0	17	-	-	1	-	-	-	5,993	6,851	6,848	6,851	6,851
SEARO	Myanmar	1,791	1,791	1,791	-	-	0	-	-	-	-	-	-	1,791	1,792	1,792	1,792	1,792
SEARO	Nepal	411	411	411	-	-	-	-	-	-	-	-	-	411	571	571	571	571
SEARO	South Korea	-	-	-	-	-	-	-	-	-	-	-	-	-	734	734	734	734
WPRO	Cambodia	589	589	589	-	-	0	-	-	-	-	-	-	589	620	620	620	620
WPRO	Laos	262	262	262	-	-	-	-	-	-	-	-	-	262	278	278	278	278
WPRO	Malaysia	1,055	1,055	1,055	-	-	-	-	-	-	-	-	-	1,055	1,056	1,056	1,056	1,056
WPRO	North Korea	-	-	-	-	-	-	-	-	-	-	-	-	-	757	757	757	757
WPRO	Papua New Guinea	279	251	292	18	6	45	1	-	3	-	-	-	298	343	343	343	343
WPRO	Philippines				-	-		-	-	-	-	-						

WHO Region	Country	*P. falciparum*													*P. vivax*			
		**Hypoendemic***	**Lower UI**	**Upper UI**	**Mesoendemic***	**Lower UI**	**Upper UI**	**Hyperendemic***	**Lower UI**	**Upper UI**	**Holoendemic***	**Lower UI**	**Upper UI**	**Overall**	**Hypoendemic***	**Lower UI**	**Upper UI**	**Overall**
		159	159	159			0						-	159	113	113	113	113
WPRO	Solomon Islands	31	29	32	1	-	3	-	-	0	-	-	-	32	32	32	32	32
WPRO	Vanuatu	13	13	13	-	-	-	-	-	-	-	-	-	13	13	13	13	13
WPRO	Vietnam	1,356	1,356	1,356	-	-	0	-	-	-	-	-	-	1,356	1,540	1,540	1,540	1,540

Parasite Rate in 2–10-Year-Olds for P. falciparum and 1–99-Year-Olds for P. vivax: 0-10%=hypoendemic; 10-50%=mesoendemic; >50%=hyperendemic; and ≥75%=holoendemic.

AFRO=Regional Office for Africa. AMRO=Regional Office for the Americas. EMRO=Eastern Mediterranean Regional Office. EURO=Regional Office for Europe. SEARO=Regional Office for South-East Asia. WPRO=Regional Office for the Western Pacific.

Different methods have been used by MAP to derive overall estimates and estimates by endemicity strata. As a result, the sum of the endemicity strata is not always equivalent to the overall estimate. See methods
